# The effect of median household income on the prognosis of lung adenocarcinoma: A SEER analysis

**DOI:** 10.1097/MS9.0000000000002488

**Published:** 2024-09-04

**Authors:** Ruina Wang, Xiaofan Lai, Binying Huang, Xiaohua Ning, Tingting Zhang

**Affiliations:** The Twelfth Guangzhou City People’s Hospital, Guangzhou, Guangdong Province, China

**Keywords:** lung adenocarcinoma, survival, mortality, income

## Abstract

**Background:**

Lung cancer is the main cause of death related to malignant tumors. Since cause-specific mortality can guide clinical decision-making, this study employed the Fine–Gray model based on the Surveillance, Epidemiology, and End Results (SEER) database to identify significantly how socio-economic status influences initial treatment decisions and survival outcomes in patients with lung adenocarcinoma.

**Objective:**

The aim of this study was to identify the predictors of lung adenocarcinoma.

**Methods:**

The U.S. Surveillance, Epidemiology, and End Results (SEER) database was used to identify patients diagnosed with lung adenocarcinoma between 2000 and 2018. Seventeen thousand four hundred forty-one patients with lung adenocarcinoma were subdivided in four socio-economic tertiles, based on median household income. Cox regression modeling explored the relationship between race, surgery, grading, and median household income on survival outcomes. The study also assessed patient demographic characteristics, age at diagnosis, and surgical interventions.

**Results:**

Among 17 441 patients with primary lung adenocarcinoma, the age distribution was as follows: less than 45 years (*n*=202, 1.16%), between 45 and 54 years (*n*=1121, 6.43%), 55 and 64 years (*n*=4252, 24.38%), 65 and 74 years (*n*=6357, 36.45%), 75 and 84 years (*n*=4426, 25.38%), and more than 84 years (*n*=1083, 6.2%). The adjusted hazard ratio (aHR) with 95% CI for ages 65–74 years, 75–84 years, and older than or equal to 85 years were 0.25 (0.11,1.29), 0.40 (0.11,1.50), and 0.72 (0.11,2.05), respectively. Multifactorial Cox regression indicated that the aHR for tumor metastasis was 0.93 (0.03, 2.54), and for patients who did not undergo surgery, it was 1.46 (0.03, 4.31). Grade IV patients exhibited the lowest survival rate [0.66(0.11, 1.93)]. A notable correlation existed between median household income and survival, with distinctly lower survival rates observed in low-income groups.

**Conclusion:**

Older patients, especially those who did not undergo surgery and had a higher tumor grade, had significantly reduced survival. Moreover, survival rates for black patients in lower-income brackets were worse than those for white patients in the same financial category.

**Implications for practice:**

The quality of life of lung cancer patients is affected by low-income family.

## Background

Highlights

- Lung cancer is the predominant cause of cancer and cancer-related deaths globally. There are two primary types of lung cancer: small cell lung cancer (SCLC) and non-small cell lung cancer (NSCLC). Notably, NSCLC constitutes ~85% of all cases. The 5-year survival rate for NSCLC patients fluctuates between 10 and 20%.
- Most patients present with an unfavorable prognosis as they are often diagnosed at an advanced or metastatic stage, rendering surgical treatment unfeasible.
- Socio-economic status, frequently gauged by median household income, emerges as another pivotal determinant.
- Since cause-specifc mortality can guide clinical decision-making, this study employed the Fine–Gray model based on the Surveillance, Epidemiology, and End Results (SEER) database to identify significantly how socio-economic status influences initial treatment decisions and survival outcomes in patients with lung adenocarcinoma.
- Most of the data in the SEER database is freely available to the public, and researchers can use it by signing relevant agreements. The data can be downloaded through the SEER*Stat software, providing extensive access to cancer research, especially for rare cancers.

Lung cancer is the predominant cause of cancer and cancer-related deaths globally^1^. The Global Cancer Epidemiology Database Report 2020 indicates that lung cancer has the highest mortality rate in females and ranks second in incidence, whereas in males, it leads both in incidence and mortality^2^. There are two primary types of lung cancer: small cell lung cancer (SCLC) and non-small cell lung cancer (NSCLC). Notably, NSCLC constitutes ~85% of all cases^3^, with lung adenocarcinoma (LUAD) being the most prevalent subtype. Current NSCLC treatments encompass surgical resection, conventional or stereotactic radiation therapy, chemotherapy, targeted therapy, and immunotherapy. However, the 5-year survival rate for NSCLC patients fluctuates between 10 and 20%^4–6^.

The prognostic factors of LUAD are still controversial. Previous studies are often based on the cohort statistical analysis of a small number of people, and the prognosis results are quite different. Even experienced clinicians still have great challenges in predicting the survival time of patients. Moreover, there are differences in medical technology among different medical institutions, which brings greater challenges to the prognosis prediction of LUAD patients. It is very important to use the clinical data of patients to build an accurate tool to predict the survival probability. Physicians and patients will benefit from a readily available and intuitive predictive model tool that can assess survival outcomes through demographic, histopathology, and surgical approaches in clinical practice.

The Surveillance, Epidemiology, and End Results (SEER) database is a cancer population registry in the United States that collects basic patient information, clinicopathological characteristics, and treatment-related data covering nearly one-third of the U.S. population^7^. In this study, we screened prognostic risk factor variables for EPN patients for statistical analysis using the SEER database and Cox regression modeling explored the relationship between race, surgery, grading, and median household income on survival outcomes.

## Methods

### Search strategy and inclusion criteria

The data utilized for the study were sourced from The Surveillance, Epidemiology, and End Results (SEER) database, established by the National Cancer Institute of the National Institutes of Health in 1973. The database has since grown to include 18 registry centers, covering an estimated 34.6% of the U.S. population, solidifying its reputation as one of the most comprehensive large-scale tumor databases in North America. The SEER database is updated annually, and the submitted data undergoes quality control and integrity checks in November of the submission year, and is made available for use in April of the following year. Most of the data in the SEER database is freely available to the public, and researchers can use it by signing relevant agreements. The data can be downloaded through the SEER*Stat software, providing extensive access to cancer research, especially for rare cancers. Moreover, the database extensively captures cancer incidence, prevalence, mortality, and other relevant medical information from selected U.S. continental counties. This includes demographics, primary tumor site, histologic type, cancer stage at diagnosis, the first course of treatment received by the patient, cause of death, and survival time^8^. The current study has been reported in line with the STROCSS criteria^9^.

### Study design

Data from the SEER database (Version: 8.4.2; https://seer.cancer.gov/data-software/), SEER Research Plus Data, 18 Registries,

March 2023 Sub (2000–2018) (username for log in:24325-Nov2021) were screened. The criteria included data for patients with LUAD coded 8140 in the International Classification of Diseases for Oncology, third edition (ICD-O-3), where the primary site for the thyroid gland was denoted as C34.1-C34.9.

The study was approved by the institutional research ethics committee. It incorporated 10 variables. These encompassed patients’ racial groups, segmented as White (non-Hispanic White), Black (non-Hispanic Black), Hispanic, and Other. Tumor grades were classified as: Well-differentiated/Grade I; Moderately differentiated/Grade II; Poorly differentiated/Grade III; Undifferentiated; anaplastic/ Grade IV.

Static county attributes (SCAs) are validated measures to assess socio-economic characteristics for patients in the SEER database^10^. SCAs are calculated every 5 years and available in SEER, allowing for the assessment of outcomes based on sociodemographic factors. A patient’s median household income (MHI) was defined as the 2010–2014 SCA estimate for the median household income of the patient’s county of residence. The median household income of patients was categorized into quartiles^11^: lowest (earning <$35 000), low ($35 000–$54 999), middle ($55 000–$54 999), and middle ($55 000–$54 999), middle ($55 000–$74 999), and highest (earning $75 000 and above).And intra-group comparison according to median household income. Lastly, the study considered whether the tumor had metastasized and if the patient underwent surgery.

Public access to the cancer research data is freely available, provided a data use agreement is executed. Importantly, acquiring data from the SEER database does not mandate patient informed consent, as the database omits any identifiable patient-specific information.

### Statistical analysis

The Kaplan–Meier method was employed for univariate survival analysis, and the log-rank test compared survival rates between groups. The univariate Cox proportional risk model assessed the relationship between individual study variables and the risk of death from lung adenocarcinoma. Simultaneously, the multivariate stratified Cox proportional risk model identified the mortality risk associated with prognostic factors in the United States after adjusting for known prognostic factors. Models were stratified by median household income to consider survival patterns over time. Hazard ratio (HR) and adjusted hazard ratio (aHR) were reported separately for both univariate and multivariate models, accompanied by 95% CIs and *P* less than 0.05 for *p* value two-sided tests. Logistic analysis identified significant factors, providing the odds ratio (OR) for determining elements influencing survival in lung adenocarcinoma patients. Data analysis was conducted using statistical software R (version 4.2.2).

## Results

**Figure 1:**
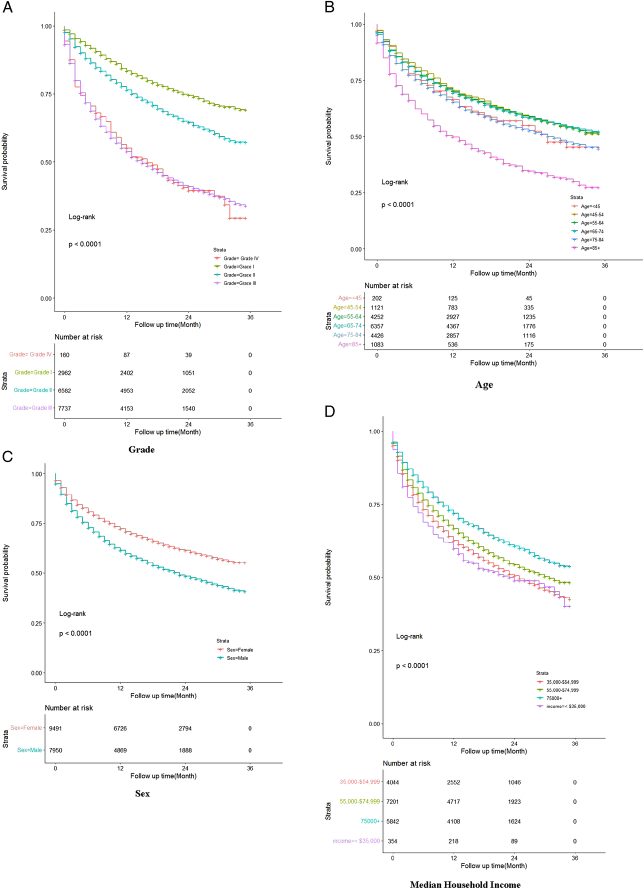
Kaplan–Meier Plots about survival rates of different factors.

The study encompassed 17 441 patients diagnosed with primary lung adenocarcinoma between 2000 and 2018. Table 1 presents an analysis of patient characteristics stratified by median household income. Age distribution was: younger than 45 years (*n*=202, 1.16%), 45–54 years (*n*=1121, 6.43%), 55–64 years (*n*=4252, 24.38%), 65–74 years (*n*=6357, 36.45%), 75–84 years (*n*=4426, 25.38%), and older than or equal to 85 years (*n*=1083, 6.2%). Based on their county of residence’s median household income (MHI), patients were categorized into four groups: 354 in the lowest (<$35 000), 4044 in low ($35 000–$54 999), 7201 in middle ($55 000–$74 999), and 5842 in highest (≥ $75 000). Kaplan–Meier curves are plotted in the Fig. 1, including the age at diagnosis, sex, Grade, and median household income.

**Table 1:** Analysis of patient characteristics stratified by median household income.

Variables	Lowest	Low	Middle	Highest
	(< $35 000)	($35 000–$54 999)	($55 000–$74 999)	(≥$75 000)
Sex, *N* (%)				
Male	190 (1.09)	2006 (11.50)	3256 (18.67)	2498 (14.32)
Female	164 (0.94)	2038 (11.69)	3945 (22.62)	3344 (19.17)
Year of diagnosis, *N* (%)				
2016	198 (1.14)	2102 (12.05)	3750 (21.50)	2890 (16.57)
2017	156 (0.89)	1942 (11.13)	3451 (19.79)	2952 (16.93)
Race, *N* (%)				
White	299 (1.71)	3219 (18.46)	5745 (32.94)	4511 (25.86)
Black	52 (0.3)	748 (4.29)	811 (4.65)	394 (2.26)
Other	3 (0.02)	77 (0.44)	645 (3.7)	937 (5.37)
Age, *N* (%)				
<45	2 (0.01)	49 (0.28)	84 (0.48)	67 (0.49)
45–54	34 (0.19)	327 (1.87)	431 (2.47)	329 (1.88)
55–64	103 (0.59)	1130 (6.48)	1738 (9.97)	1281 (7.34)
65–74	144 (0.83)	1474 (8.45)	2605 (14.94)	2134 (12.24)
75–84	62 (0.36)	891 (5.02)	1877 (10.76)	1596 (9.15)
85+	9 (0.05)	173 (0.99)	466 (2.67)	435 (2.49)
Stage, *N* (%)				
Yes	109 (0.63)	1388 (7.96)	2460 (14.10)	2301 (13.19)
No	245 (1.40)	2656 (15.23)	4741 (27.18)	3541 (20.30)
Surgery, *N* (%)				
Yes	127 (0.73)	1632 (9.36)	2917 (16.73)	2690 (15.42)
No	227 (1.30)	2412 (13.83)	4284 (24.56)	3152 (18.07)
Grade, *N* (%)				
Grade I	32 (0.18)	618 (3.54)	1174 (6.73)	1138 (6.52)
Grade II	133 (0.77)	1518 (8.7	2769 (15.88)	21629 (12.40)
Grade III	188 (1.08)	1877 (10.76)	3196 (18.32)	2476 (14.20)
Grade IV	1 (0.01)	31 (0.18)	62 (0.36)	66 (0.37)

The Cox regression analysis explored the influence of various factors on the survival of patients with lung adenocarcinoma. The results, commonly interpreted through HR, indicated that all effects were significant (*P*<0.05). Older patients exhibited a slightly elevated mortality risk compared to their younger counterparts. The aHR (95% CI) was 0.25 (0.11, 1.29) for ages 65–74 years, 0.40 (0.11, 1.50) for 75–84 years, and 0.72 (0.11, 2.05) for those over 84 years. A notable difference in mortality risk was observed between patients with metastatic tumors and those who did not undergo surgery, with an aHR (95% CI) of 0.93 (0.03, 2.54) for no surgery and 1.46 (0.03, 4.31) for metastatic tumors.

## Discussion

The study employed Cox regression modeling and Kaplan–Meier survival to shed light on the various factors influencing the survival of patients with lung adenocarcinoma.

The table reveals that the incidence of lung adenocarcinoma was lowest in those under 45 years and peaked in the 65–74 year age group. The survival rate of tumor patients is inversely related to the age of diagnosis; the older the age at diagnosis, the lower the long-term survival rate. This observation aligns with both domestic and international studies^12^. Age at diagnosis is a crucial indicator of patient survival. Our findings underscore the importance of factoring in both age and age at diagnosis when making clinical and prognostic assessments for patients with lung adenocarcinoma. Younger patients, diagnosed at an earlier age, often exhibit higher survival rates. This disparity might stem from the superior physical health and resilience of younger patients at diagnosis, leading to more frequent surgeries. Conversely, older patients might opt for more conservative treatments due to pre-existing health conditions and simultaneous health issues^13^.

The study found that the incidence of lung adenocarcinoma was higher in females than in males. Numerous epidemiological studies have identified a distinct correlation between gender and lung cancer risk^14,15^. According to existing literature, females have a heightened risk of developing lung cancer^15^, potentially due to specific risk factors such as hormonal dynamics or increased susceptibilities^16^. Nonetheless, the survival rate for lung adenocarcinoma was observed to be higher in women compared to men in the study.

The study revealed that treatment modality significantly impacts the prognosis of patients with lung adenocarcinoma. While surgery is the primary treatment choice for GBM, its aim is to directly excise tumor lesions, thus inhibiting tumor growth and dissemination. Complete resection is the foundational treatment for patients with benign tumors that have distinct margins and are easier to remove. This approach can effectively eliminate the lesions and notably extend patients’ survival time. However, for cancer patients with high malignancy or lymphocyte metastasis, such surgery can harm surrounding vascular tissues, increasing the likelihood of post-surgical recurrence. For these patients, a combination of partial resection, radiation therapy, or chemotherapy may be more appropriate. It has been demonstrated that a surgical-based treatment modality, complemented by other therapeutic methods, can extend patient lifespan and enhance their quality of life^13^.

Survival rates vary according to socio-economic status. Poverty is inversely related to survival, with patients of higher socio-economic standing experiencing a relatively better survival rate. Economic capability often dictates whether treatment is obtained, how it is delivered, and insurance choices. A study from Australia and the United States discovered a stronger correlation of morbidity risk with higher socio-economic status^14^. However, U.S. studies have indicated that individuals with lower socio-economic status receive less social support, experience diminished social cohesion, and have reduced access to material resources and healthcare services. These factors might be more pivotal in determining mortality risk^17^. Individuals in higher income brackets might have better access to family doctors or be more proactive in seeking timely medical attention, leading to earlier diagnoses and treatment, thereby improving survival rate^18^. Conversely, those in lower-income groups might delay seeking medical care. For instance, they might only approach healthcare providers when symptoms become pronounced. They may lack consistent access to a family doctor, resulting in postponed health evaluations and subsequent treatments^19^, causing delays in diagnosis and treatment, exacerbating the illness, and decreasing survival rates.

These results should be interpreted considering several limitations. The study’s reliance on retrospective data means it cannot fully account for potential confounders like environmental, nutritional, and psychosocial factors. The data were sourced from the SEER database, which does not provide information on radiotherapy, chemotherapy, or radiochemotherapy. While the SEER database’s data on lung adenocarcinoma might be relevant globally, the majority of the information is from the United States, limiting its general applicability.

## Conclusion

Our data show that a lower median household income is associated with quality of lung adenocarcinoma survival. Some factors likely contribute to this inequality, including inadequate communication, misaligned perceptions of care, long distances from referral centers, and financial concerns. Further research is needed to evaluate the root causes and barriers to access for patients with LUAD from low SES backgrounds to influence policymaking and quality improvement measures. Finally, National medical insurance should be more inclined to the family funds of low-income cancer patients, such as family care and study for children.

### Limitations

This study uses data from the United States, and the number of white patients is too large. Therefore, it is necessary to pay attention to racial and cultural differences in the promotion and use.

### Implications for practice

Lung cancer is very common. The postoperative survival time was prolonged. In order to improve the quality of life, it is necessary to understand the influencing factors and start from a multi-dimensional perspective. Nurses play an important role in developing and promoting interventions to improve quality of life.

## Ethical approval

The patient data involved came from The U.S. Surveillance, Epidemiology, and End Results (SEER) database.

## Consent

The data utilized for the study were sourced from The Surveillance, Epidemiology, and End Results (SEER) database, established by the National Cancer Institute of the National Institutes of Health in 1973.

## Source of funding

This research was Guangzhou Municipal Science and Technology Program key projects supported, SL2024A03J00805.

## Author contribution

R.W.: study concept or design,writing the paper. X.L. and B.H.: data cleansing. X.N. and T.Z.: data analysis and manuscript reviewer. All the authors read and approved the final manuscript.

## Conflicts of interest disclosure

The authors declare no conflicts of interest in relation to this work.

## Research registration unique identifying number (UIN)

No.

## Guarantor

The Twelfth People's Hospital of Guangzhou, Guangzhou, Guangdong, China.

## Data availability statement

The data that support the findings of this study are available in the supporting information of this article.

## Provenance and peer review

Yes.
